# A Case of Late Type Ia Endoleak After Endovascular Aneurysm Sealing Using the Nellix System: Proximal Extension with Triple Chimney and Gutter Endoleak Embolization

**DOI:** 10.3400/avd.cr.21-00092

**Published:** 2021-12-25

**Authors:** Pietro Volpe, Antonino Alberti, Vittorio Alberti, Mafalda Massara

**Affiliations:** 1 Vascular and Endovascular Surgery Unit, Grande Ospedale Metropolitano “Bianchi-Melacrino-Morelli,”Reggio Calabria, Italy

**Keywords:** EVAS, type Ia endoleak, embolization

## Abstract

An 87-year-old man, who submitted to endovascular aneurysm sealing (EVAS) on 2017, presented a type Ia endoleak 2 years later, with enlargement of the aneurysmal sac. We planned an endovascular procedure of correction consisting of a proximal extension through two covered stent grafts deployed into the previous Nellix stent grafts, with associated triple chimney. However, 3 months later, he had a further 5 mm aneurysmal sac enlargement. He was submitted to angiography with coil embolization of gutters, obtaining a successfully result. At 1 and 3 months, he is free from endoleak, with a stable aneurysmal diameter.

## Introduction

Endovascular aneurysm sealing (EVAS), using the Nellix endoprosthesis (Endologix, Irvine, CA, USA), represented an alternative modality of infrarenal abdominal aortic aneurysm (AAA) endovascular repair, based on two balloon expandable covered stents surrounded by endobags filled with a soluble polymer at the time of the procedure that guarantees fixation and seal. This different technology aimed to reduce the endoleaks and subsequent reoperation rates.

However, any kind of endoleaks can occur after EVAS and in particular for type Ia endoleak diagnosis may be difficult based on computed tomography (CT) images, due to the radiopacity of endobags, especially in the first month after operation.

We present a case of late type Ia endoleak that occurred 2 years after EVAS and the strategy of treatment is discussed.

## Case Report

**Figure 1:**
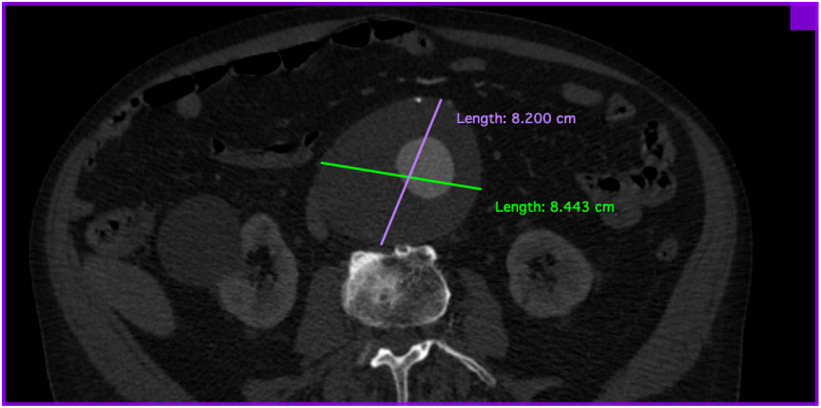
Fig. 1 Preoperative computed tomography image showing a voluminous infrarenal abdominal aortic aneurysm with a transverse diameter of 82×84 mm.

An 87-year-old man affected by hypertension, chronic obstructive pulmonary disease, and mild renal failure (estimated glomerular filtration rate=55 ml/min) was successfully submitted to EVAS on 2017 for a voluminous infrarenal AAA (82×84 mm=transverse diameter) (**Fig. 1**) and judged at high risk of type II endoleak occurrence due to patency of three couples of lumbar arteries, with a middle diameter of 2.5 mm, one of which arising at the level of renal arteries, where mural thrombus was completely absent.

**Figure 2:**
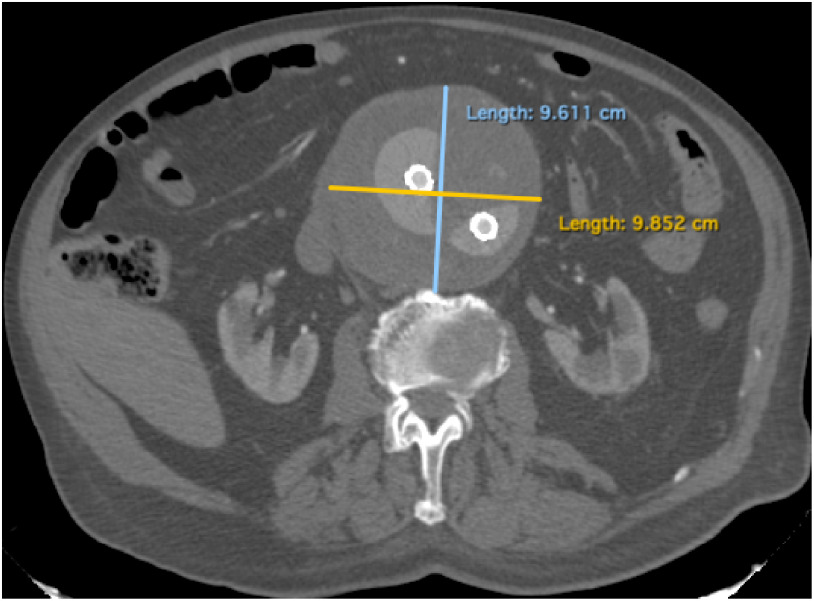
Fig. 2 Increasing transverse diameter of the aneurysmal sac 2 years after endovascular aneurysm sealing.

Due to his mild renal failure, follow-up consisted of ultrasonography at 6 months and CT scan at 12 months. During follow-up, 2 years after operation, the patient presented a sudden increase of the transverse diameter of the AAA (96×98 mm) on CT scan control (**Fig. 2**).

A careful analysis and study of the CT scan showed the presence of a type Ia endoleak, probably due to a migration of the two stent grafts of the Nellix endoprosthesis of about 4–5 mm. Open conversion was excluded because our patient was judged unfit for open surgery. A Nellix in Nellix proximal extension was excluded because the Nellix endoprosthesis was no longer available at the time of operation. A correction recurring to a custom-made endoprosthesis was excluded due to long production times in a patient with a sudden increase of the transverse diameter of the aneurysmal sac.

So, the only endovascular option to correct the problem was a proximal extension that required an associated triple chimney [a double chimney on both renal arteries (RA) and a chimney on the superior mesenteric artery (SMA)] in order to guarantee an adequate proximal sealing in an old patient unfit for open surgery and considered an urgent case to be operated as soon as possible.

Due to comorbidities, the patient underwent the procedure under local anesthesia. A bilateral retrograde percutaneous femoral artery access was required, associated with a left percutaneous brachial artery access to cannulate visceral arteries. After introducer deployment, intravenous heparin was given (5,000 UI). After an angiography to locate RA and SMA, all these vessels were cannulated from the brachial artery access and a stiff guide wire was positioned. Over this wire, sheaths (usually 6-F or 7-F) were placed to deliver Atrium Advanta V12 stents (Atrium Maquet Getinge Group, Mijdrecht, the Netherlands) into RA and SMA, and the procedure was completed with proximal extension, introducing two peripheral covered iliac limbs (W. L. Gore and Associates, Flagstaff, AZ, USA) into the two previous stent grafts of the Nellix endoprosthesis.

The final angiography showed the correct deployment of the endoprosthesis with visceral arteries stents regular patency.

**Figure 3:**
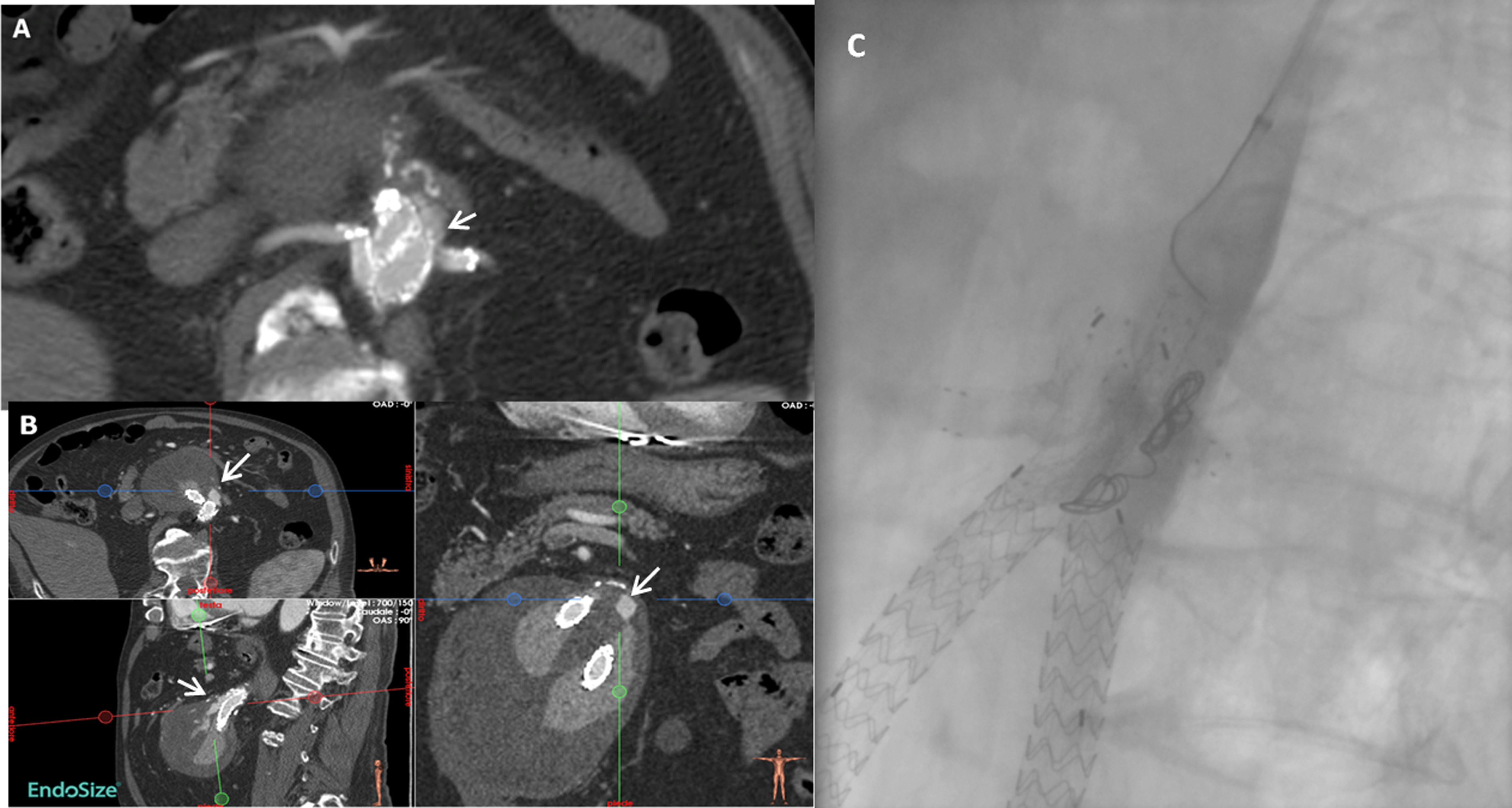
Fig. 3 Axial computed tomography (CT) image showing contrast medium between left renal artery stent and the two covered aortic stents after ChEVAS and proximal extension (**A**); persistent type Ia endoleak in different CT image views (**B**). Intraoperative angiographic image showing the final result after gutter embolization through coils (**C**).

Six months later, a CT scan control showed a further increase of the transverse abdominal aortic diameter with persistent type Ia endoleak (**Figs. 3A** and **3B**). Based on the suspicion of persistent type Ia endoleak due to gutters after the chimney procedures, the patient was submitted to an angiography that confirmed the suspicion. Under local anesthesia, a left brachial artery access was gained and a Destination Sheath (6F×90 cm, Terumo Europe, Leuven, Belgium) was placed. After angiography, a 5F Ber catheter (Cordis, Miami, FL, USA) was used to enter into endoleak and a 0.018 microcatheter (Rebar 18; Medtronic, Inc., Minneapolis, MN, USA) was placed into the endoleak to release two (16 mm 30 cm the first one and 14 mm 30 cm the second one) Concerto Helix Detachable Coil System (Medtronic, Inc., Minneapolis, MN, USA), with successful embolization of gutters (**Fig. 3C**).

One and three months later, an ultrasonographic control showed a stable transverse diameter of the AAA and the patient continued to be followed up.

## Discussion

EVAS represents an alternative method of treating AAA than the traditional endovascular aneurysm repair (EVAR).^1)^ It consists of two balloon expandable covered stents, each surrounded by endobags filled with a soluble polymer at the time of the procedure, after implantation: the amount of polymer is previously calculated, based on aneurysmal sac volume at preoperative CT scan and the job of endobags is to guarantee an adequate fixation and seal.^2)^

This endoprosthesis was designed with the aim of reducing the endoleak rate of any kind, especially type II endoleaks, and subsequently the reoperation rate during the follow-up period.^2)^

About the endoleak incidence after EVAS, the Nellix system investigational device exemption pivotal trial reported an endoleak rate of 6.3% at 30 days, in a group of 142 patients (type I, 0.7%; type II, 5.6%), with a 1-year persistent endoleak rate of 3.1% (type I, 0.8%; type II, 2.3%).^3)^

The EVAS FORWARD Global Registry comprising 277 patients reported an early type Ia endoleak in eight cases,^4)^ probably due to an inadequate use of the proximal seal zone and an underfilling of the endobags.

As reported, the published incidence of endoleak is low in the short-term, but mid-term results are not encouraging. In addition, the management especially of proximal endoleaks and migration differs from those after conventional EVAR, precisely due to the specific structural characteristics of the endoprosthesis.

Also, the diagnosis of complications after EVAS should be difficult respect to traditional EVAR for the presence of the endobags. Postoperative CT scan images are different and change with time and also complications have a different CT aspect. In particular, the radio density of the polymer within endobags is usually high early after implantation, rendering the early diagnosis of type Ia endoleak difficult.^5)^

With time, there is a progressive migration of contrast medium to the margins of the hydrogel polymer within the endobags and so the endobag aspect changes. On CT scan, type I endoleak usually presents as a curvilinear area of flow between the endobag and the aortic wall.^5)^

Regarding type Ia endoleak, some authors reported a particular classification distinguishing four types, depending on the location of the contrast medium for the first three types, while the fourth type is characterized by sac pressurization, in the absence of evident signs of endoleak.^2)^

Based on this classification, optimal treatment modalities for type Ia endoleaks post-EVAS vary according to the type of endoleak.^2)^

Type Is1 endoleak treatment aims to improve the proximal seal: any gutters present can be obliterated with coils in combination with Onyx or glue.^6,7)^

For type Is2 endoleak resolution, if the two stents are correctly located, embolization with coils and Onyx/glue can be used. In cases of distal migration or malpositioning of the two covered stents, a proximal Nellix-in Nellix extension is required to obtain an adequate proximal seal,^8)^ with a sealing zone of 20 mm in cases of chimney and of at least 30 mm in the other cases.

Type Is3 endoleak treatment requires a Nellix-in Nellix proximal extension associated or not with chimney grafts, in order to obtain a proximal adequate seal.

Type Is4 endoleak generally does not require immediate treatment but a careful monitoring and observation, with operation only in cases of disease progression.

According to these authors,^2)^ open surgical repair may be necessary for types Is2 and Is3, especially after endovascular option failure, while in a more recent study about 101 patients submitted to EVAS, a type Ia endoleak occurred on 19.8% and the first strategy of treatment consisted of open conversion on 70% of cases, considering proximal extension and embolization as a possible alternative to open conversion.^9)^

Our patient presented a late type Is2 endoleak probably due to a migration of about 5 mm of the two covered stent grafts, with subsequent aneurysmal sac enlargement from 84 mm (before operation) to 98 mm, judged at high risk of rupture. Due to severe comorbidities, we opted for an endovascular correction recurring to proximal extension through two covered stents and triple chimney. However, our patient had persistent type Ia endoleak from gutters, which required reintervention and a successful treatment recurring to coil embolization.

## Conclusion

Even if early results after EVAS were promising, mid-term results were not encouraging, for the high rate of complications. Respect to traditional EVAR, in particular for type Ia endoleaks as reported for our patient, treated with proximal extension associated with triple chimney and finally with successful gutter endoleak embolization.
